# Analysis of the Domains of Hepatitis C Virus Core and NS5A Proteins that Activate the Nrf2/ARE Cascade

**Published:** 2016

**Authors:** O. A. Smirnova, O. N. Ivanova, F. Sh. Mukhtarov, V. L. Tunitskaya, J. Jansons, M. G. Isaguliants, S. N. Kochetkov, A. V. Ivanov

**Affiliations:** Engelhardt Institute of Molecular Biology, Russian Academy of Sciences, Vavilov str., 32, Moscow, 119991, Russia; Riga Stradins University, Dzirciema Street, 16, LV-1007, Riga, Latvia; N. F. Gamaleya Research Center of Epidemiology and Microbiology, Ministry of Health of the Russian Federation, Gamaleya str., 18, Moscow, 123098, Russia

**Keywords:** Hepatitis C virus, oxidative stress, regulation, transcription factor, Nrf2

## Abstract

The hepatitis C virus (HCV) triggers a chronic disease that is often accompanied by a spectrum of liver pathologies and metabolic alterations. The oxidative stress that occurs in the infected cells is considered as one of the mechanisms of HCV pathogenesis. It is induced by the viral core and NS5A proteins. It is already known that both of these proteins activate the antioxidant defense system controlled by the Nrf2 transcription factor. Here, we show that this activation is mediated by domain 1 of the NS5A protein and two fragments of the core protein. In both cases, this activation is achieved through two mechanisms. One of them is mediated by reactive oxygen species (ROS) and protein kinase C, whereas the other is triggered through ROS-independent activation of casein kinase 2 and phosphoinositide 3-kinase. In the case of the HCV core, the ROS-dependent mechanism was assigned to the 37–191 a.a. fragment, while the ROS-independent mechanism was assigned to the 1–36 a.a. fragment. Such assignment of the mechanisms to different domains is the first evidence of their independence. In addition, our data revealed that intracellular localization of HCV proteins has no impact on the regulation of the antioxidant defense system.

## INTRODUCTION

The hepatitis C virus (HCV) is a widespread and dangerous pathogen that infects the human liver. In most cases, HCV infection leads to chronic hepatitis, during which there is a high risk of liver fibrosis and cirrhosis, hepatocellular carcinoma, and various metabolic disorders (steatosis, type 2 diabetes mellitus, altered iron metabolism and other pathologies) [1]. Numerous basic and clinical studies have revealed a number of pathogenetic mechanisms related to HCV, including oxidative stress (OS), which plays an important role [2]. OS is a state of a cell characterized by an imbalance between reactive oxygen species (ROS) and the low-molecular- weight compounds (antioxidants) that neutralize them, as well as the enzymes involved in the protection against ROS (referred to as phase II enzymes). The biosynthesis of many enzymes of the antioxidant metabolism and phase II enzymes is controlled by the Nrf2 transcription factor (nuclear factor-erythroid 2-related factor 2), which binds to the common regulatory element ARE (Antioxidant Response Element) in gene promoters [3]. In the absence of stress, the Nrf2 factor is located in the cytoplasm in a complex with its partner protein Keap1. When ROS production is up-regulated, this complex is disrupted either through phosphorylation of the Nrf2 factor by various protein kinases or as a result of Keap1 modification, the free transcription factor is then translocated to the nucleus and transcription with a concomitant activation of the genes encoding antioxidant defense such as heme oxygenase-1 (HO-1) and NAD(P)H:quinone oxidoreductase 1 (Nqo1) [3, 4].

The hepatitis C virus does not only cause OS, but it also activates the transcription factor Nrf2 [2]. In both cases, the viral core and NS5A proteins play the key role [4-7]. Previously, we established that these proteins activate the defense system through two mechanisms, one of which is mediated by ROS and protein kinase C, and the other through ROS-independent activation of factor Nrf2 by casein kinase 2 and phosphoinositide 3-kinase [4]. However, it cannot be excluded that both pathways of Nrf2 activation are induced by some common regulator located earlier in the cascade, all the members of which remain unknown. The objective of the current study was to reveal the presence (or absence) of this regulation and identify the structural elements of the core and NS5A proteins involved in the mechanisms of Nrf2/ARE cascade activation.

## EXPERIMENTAL SECTION

A human hepatoma Huh7.5 cell line was provided courtesy of C. Rice (Rockefeller University, USA). The plasmid pCMV-core encoding the full-length core protein of HCV genotype 1b (274933RU) has been previously described [4]. Plasmids encoding fragments 1–36, 37–191, and 1–151 a.a. of the core protein were constructed on the basis of the pVax1 vector [8], plasmids encoding the full-length protein NS5A of HCV genotype 1b (AJ238799) and its individual D1 domains (residues 1–249), D2 (250–355 a.a.), and D3 (356–447 a.a.) were based on the pCMV-Tag3B vector [7].

Ro 31-8220, wortmannin, and 5,6-dichloro-1-β-*D*ribofuranosylbenzimidazole (DRB) (Sigma) were used as inhibitors of the protein kinases.

**Culture procedures**

Huh7.5 cells were cultured in a DMEM medium supplemented with 10% fetal bovine serum (HyClone, USA), 2 mM glutamine, 50 u/ml penicillin, and 50 μg/ml streptomycin at 37°C in humidified atmosphere of 5% CO_2_.

**Work with reporter plasmid**

Huh7.5 cells were seeded in 24-well plates, transfected with a mixture of reporter plasmid pP-ARE (0.25 μg) [4] and a target-expressing plasmid (0.25 μg) using the Turbofect reagent. After 30 hours, the cells were lysed and luciferase activity was measured as previously described [4].

**Reverse transcription and real-time PCR (RT-qPCR)**

The Huh7.5 cells were transfected as described above. Forty hours posttransfection, the culture medium was collected, total RNA isolated, and reverse transcription and real-time PCR were performed as previously reported [4].

**Western blotting**

The Huh7.5 cells were transfected in 6-well plates and lysed 40 h posttransfection. Further manipulations were performed as previously described [4] using mouse monoclonal antibodies against heme oxygenase 1 (ab13248), NAD(P)H:quinone oxidoreductase 1 (ab28947) and β-actin (ab3280) (Abcam, UK) and also secondary antibodies against mouse IgG conjugated with horseradish peroxidase (sc-2005) (Santa-Cruz, USA). For the analysis of the intracellular localization of the Nrf2 factor, the cells were lysed and fractions of cytoplasmic and nuclear proteins were separated using a commercial NE-PER kit (Thermo Scientific). Nrf2 was detected in each fraction by immunoblotting using rabbit polyclonal antibodies against Nrf2 (sc-722) and secondary antibodies against rabbit IgG (sc-2004) (Santa-Cruz).

**Statistical analysis**

The data were processed using the StatPlus software (AnalystSoft, Canada). The results are presented as a mean ± standard deviation. The statistical significance of the differences was calculated using the paired Student’s* t-*test.

## RESULTS AND DISCUSSION

**Fig. 1:**
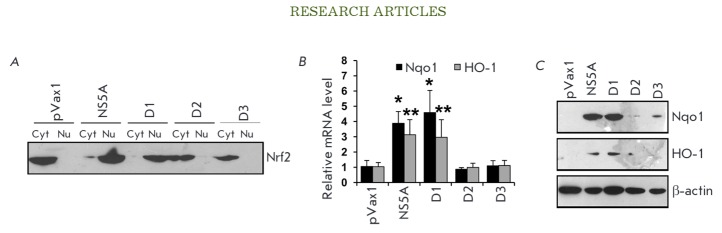
Activation of the Nrf2/ARE cascade by domain 1 of the NS5A protein results in the translocation of the Nrf2 transcription factor from the cytoplasm into the nucleus (*A*) and induction of human NAD(P)H:quinone oxidoreductase 1 (Nqo1) and heme oxygenase 1 (HO-1) (*B, C*). Intracellular localization of factor Nrf2 was determined by separation of cytoplasmic (Cyt) and nuclear (Nu) protein fractions, with subsequent detection of Nrf2 by immunoblotting. Quantification of the Nqo1 and HO-1 expression levels was performed by reverse transcription and real-time PCR (*B*) and immunoblotting (*C*). * p < 0.01 and **p  < 0.05 compared to pVax1.

Involvement of the fragments of the core and NS5A proteins in the activation of the Nrf2/ARE cascade was analyzed by three methods: quantification of the relative expression levels of the two phase II enzymes (Nqo1, HO-1) by western blotting and RT-qPCR and identification of the intracellular localization of the Nrf2 factor. It has been demonstrated that, among all of the described NS5A protein domains, only domain 1 (1–249 a.a.) can activate the Nrf2 factor; *i.e., *it causes its translocation from the cytoplasm to thenucleus (*Fig. 1A*) and enhances the expression of Nrf2-dependent genes (*Fig. 1B,C*). It is noteworthy that domain 1, among all of the three domains of the NS5A protein, exhibits a specific three-dimensional structure [9], while domains 2 and 3 are unstructured [10, 11]. Our previous data also indicate that domain 1 possesses prooxidant activity [7]. Furthermore, it has been shown that the ability of the NS5A protein to activate the Nrf2/ARE cascade is associated neither with its posttranslational modification (phosphorylation of domains 2 and 3) [12] nor with the ability to disrupt the expression of the interferon β response to HCV infection [1].

**Fig. 2:**
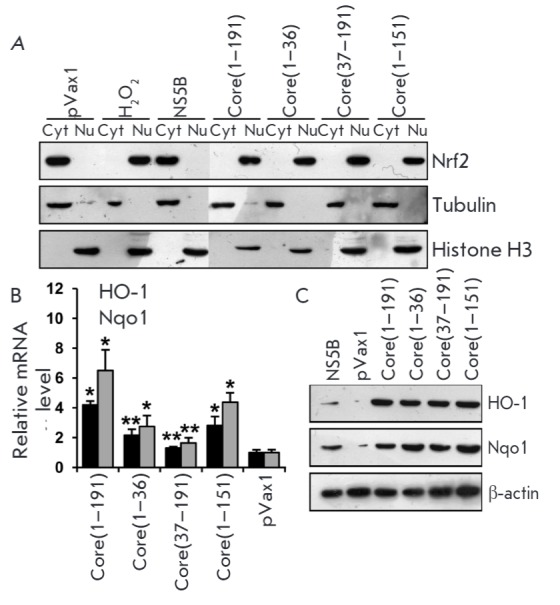
Fragments 1–36 and 37–191 a.a. of the HCV core protein activate the Nrf2/ARE cascade by triggering the translocation of transcription factor Nrf2 from the cytoplasm into the nucleus (*A*) with subsequent induction of human NAD(P)H:quinone oxidoreductase 1 (Nqo1) and heme oxygenase 1 (HO-1) (*B, C*). Intracellular localization of factor Nrf2 was determined by separation of cytoplasmic (Cyt) and nuclear (Nu) protein fractions, with subsequent detection of Nrf2 by immunoblotting. Quantification of the Nqo1 and HO-1 expression levels was performed by reverse transcription and real-time PCR (*B*) and immunoblotting (*C*). *p < 0.01 and **p < 0.05 compared to pVax1.

In order to study the contribution of various fragments of the core protein (residues 1–191 a.a.) in the activation of the Nrf2/ARE cascade, we used its truncated fragments 1–36 and 37–19 a.a. that previously were shown to trigger ROS production through a variety of mechanisms [8]. Moreover, we used the 1–151 a.a. fragment, which activated all ROS-producing enzymes as the full-length HCV despite being localized not on the endoplasmic reticulum but in the nucleus, as the 1–36 a.a. form does. It was found that all the truncated forms of the HCV core activate the Nrf2 factor (*Fig. 2A*) and induce Nrf2-dependent genes (*Fig. 2B,C*). Thus, there are at least two regions in the core protein that activate the Nrf2/ARE cascade.

**Fig. 3:**
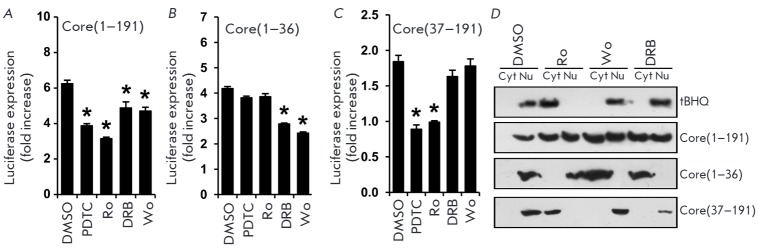
Fragment 1–36 a.a. of the HCV core protein activates the Nrf2/ARE cascade by the ROS-independent mechanism involving casein kinase 2 and phosphoinositide 3-kinase; the 37–191 a.a. fragment – by the ROS-dependent mechanism involving protein kinase C. The role of protein kinases and reactive oxygen species was estimated by measuring luciferase expression in Huh7 cells co-transfected with the reporter pP-ARE plasmid with constructs encoding the full-length core protein (*A*) or its fragments 1–36 a.a. (*B*) and 37–191 a.a. (*C*) or by an analysis of the intracellular localization of Nrf2 (*D*). The latter was studied by separation of cytoplasmic (Cyt) and nuclear (Nu) protein fractions, with subsequent detection of Nrf2 by immunoblotting. Inhibitors of protein kinases Ro 31-8220 (Ro, protein kinase C inhibitor), DRB (casein kinase 2 inhibitor), or wortmannin (Wo, phosphoinositide 3-kinase inhibitor) in the absence or presence of an antioxidant pyrrolidine dithiocarbamate (PDTC) were added into the culture medium 18 h post-transfection. *p < 0.01.

Several groups of researchers have reported that the Nrf2/ARE cascade can be activated by various protein kinases, including protein kinase C, casein kinase 2, phosphoinositide 3-kinase, the mitogen-activated protein kinases p38, ERK1/2 and JNK, or regulated by glycogen synthase kinase 3 (GSK3), with the contribution of each kinase being dependent on the cell type and stimulus ([3, 4] and references therein). In order to determine the activation mechanism for each protein fragment, we used antioxidant pyrrolidine dithiocarbamate (PDTC), as well as inhibitors of protein kinase C (Ro 31-8220, Ro), casein kinase 2 (DRB), and phosphoinositide 3-kinase (wortmannin, Wo): *i.e. *the enzymes that are, according to our data, involved in the activation of Nrf2 by the full-length NS5A protein [4]. Using a reporter plasmid pP-ARE encoding luciferase under the control of the minimum ARE-Nqo1 element of the human *Nqo1 *gene [4], we verified that both the 1–36 and 37–191a.a. fragments of the HCV core activate the transcription of ARE-dependent genes. Treatment with the antioxidant or the protein kinase C inhibitor reduced the level of luciferase expression in the case of the full-length core protein (*Fig. 3A*) and prevented activation in the case of the 37–191 a.a. fragment (*Fig. 3B*). In cells expressing the N-terminal fragment, luciferase expression was blocked only by the inhibitors of casein kinase and phosphoinositide 3-kinase (*Fig. 3B*). Similar results were obtained when studying the action of protein kinase inhibitors on the translocation of the Nrf2 factor (*Fig. 3D*). It is noteworthy that in the case of the full-length core protein, the inhibitors of all three protein kinases failed to completely prevent Nrf2 translocation to the nucleus and could only inhibit the process by approximately 2-fold, indicating a comparable contribution of the two observed mechanisms in the activation of the cascade.

Our findings showing that the N-terminal domain of the HCV core protein activates Nrf2 through a ROSindependent mechanism involving casein kinase 2 and phosphoinositide 3-kinase, while the fragment 37–191 acts through the ROS-dependent pathway involving protein kinase C, allowed us to confirm the complete independence of these two mechanisms. Moreover, casein kinase 2 and phosphoinositide 3-kinase were activated by the same domain of the HCV core that had been previously shown to interact with various proteins of the host cell, including helicase DDX3, the STAT1 transcription factor and lymphotoxin β receptor ([1, 8] and references therein). In addition, both mechanisms of Nrf2/ARE cascade activation were triggered by different variants of the core protein that are localized in the nucleus (fragments 1–36 and 1–151 a.a.) and on the surface of the endoplasmic reticulum (fragments 37– 191 and 1–191 a.a.). Therefore, it is tempting to speculate that activation of the cascade could be achieved during the biosynthesis of the core protein in the endoplasmic reticulum.

## CONCLUSIONS

In the current paper we have identified the regions of the HCV core and NS5A proteins that trigger activation of the Nrf2/ARE cascade. In addition, we have shown that the ROS-dependent and ROS-independent mechanisms of this activation are independent.

## Abbreviations

ROS: reactive oxygen species
a.a.: amino acids
HCV: hepatitis C virus
OS: oxidative stress
